# Importance of Non-Covalent Interactions in Yeast Cell Wall Molecular Organization

**DOI:** 10.3390/ijms25052496

**Published:** 2024-02-21

**Authors:** Tatyana S. Kalebina, Valentina V. Rekstina, Elizaveta E. Pogarskaia, Tatiana Kulakovskaya

**Affiliations:** 1 Department of Molecular Biology, Faculty of Biology, Lomonosov Moscow State University, Moscow 119991, Russia; vrextina@gmail.com (V.V.R.); liza.pogarskaya@gmail.com (E.E.P.); 2 Federal Research Center “Pushchino Scientific Center for Biological Research of the Russian Academy of Sciences”, Skryabin Institute of Biochemistry and Physiology of Microorganisms, Pushchino 142290, Russia; alla@ibpm.pushchino.ru

**Keywords:** yeast, cell wall, proteins, polysaccharide-remodeling enzymes, non-covalent interactions, polyphosphates, acid phosphatase

## Abstract

This review covers a group of non-covalently associated molecules, particularly proteins (NCAp), incorporated in the yeast cell wall (CW) with neither disulfide bridges with proteins covalently attached to polysaccharides nor other covalent bonds. Most NCAp, particularly Bgl2, are polysaccharide-remodeling enzymes. Either directly contacting their substrate or appearing as CW lipid-associated molecules, such as in vesicles, they represent the most movable enzymes and may play a central role in CW biogenesis. The absence of the covalent anchoring of NCAp allows them to be there where and when it is necessary. Another group of non-covalently attached to CW molecules are polyphosphates (polyP), the universal regulators of the activity of many enzymes. These anionic polymers are able to form complexes with metal ions and increase the diversity of non-covalent interactions through charged functional groups with both proteins and polysaccharides. The mechanism of regulation of polysaccharide-remodeling enzyme activity in the CW is unknown. We hypothesize that polyP content in the CW is regulated by another NCAp of the CW—acid phosphatase—which, along with post-translational modifications, may thus affect the activity, conformation and compartmentalization of Bgl2 and, possibly, some other polysaccharide-remodeling enzymes.

## 1. Introduction

The cell wall (CW) is the outer part of the yeast envelope compartment and also includes the plasma membrane and the periplasmic space. The CW completely covers the yeast cells and consists of mannoproteins—structural molecules and enzymes with different extents of glycosylation (app 35–40%), β-1,3- and β-1,6-glucan (app 55–60%) and chitin (app 1–4%) [1,2]; their minor components are lipids and polyphosphates [3,4,5].

The yeast CW is a zone of direct contact between yeast cells and the environment.

In addition to the obvious role of the external skeleton of yeast, which the maintenance of the cell shape depends on, CW is a physiologically active organelle that is involved in the complex of interactions between the microorganism and the environment. It participates in the pathways of various compounds from the environment into the cell and back [6,7,8,9,10,11,12]. CW provides resistance to external influences and is a nutrition zone where the cleavage of substrates begins [8,13]. It also plays an important role in the adaptation of these microorganisms to various carbon sources, in development and aging, in response to stress [14,15,16,17], in the regulation of morphogenesis [18] and in adhesion [19]. Receptors that receive different signals from the environment [20,21,22,23], as well as antigens that determine the immunological properties of the yeast cells, are also attached to the CW [24,25].

The structural basis of the CW is the mega-glycoconjugate (mGC) that coats the yeast cell. More than half of the mGC consists of a covalently bound network of unbranched chitin, sparsely branched molecules of highly polymeric β-1,3-glucan and branched β-1,6-glucan, with functionally important mannoproteins. The formation of the glucan network of mGC and its constant rearrangement in the process of cell growth and division are provided by the proteins that are anchored to the CW with and without forming a covalent bond with glucan [26,27,28]. 

The molecular structure of the yeast CW is very dynamic, and its remodeling occurs during budding, flocculation, mating and the transition to pseudohyphal growth [8,13,29,30,31]. The structure, and occasionally composition, of CW varies depending on the stage and conditions of culture growth, as well as the stage of the yeast cell cycle. At the same time, it is a very stable organelle, the general structure and functional capacity of which are supported by cells in various, even extreme, conditions [32,33,34,35].

The structure and methods of biosynthesis of yeast CW polysaccharides was studied in general by the 1980s [36,37,38,39,40,41,42,43,44,45,46]. After that, it became obvious that without detailed knowledge of not only polysaccharides, but also the proteins and inorganic polyphosphates (polyP) [47] that make up the cell wall, it is impossible to understand the mechanisms of its molecular ensemble formation and, ultimately, to determine how the yeast CW functions [48].

## 2. Cell Wall Proteins—Evolution of Viewpoints

At the beginning of the investigations in this field, the question of the existence of proteins that perform specific functions in the yeast CW was very controversial. Many researchers believed that the bulk of proteins of different degrees of glycosylation (mainly enzymes) isolated from CW preparations was the cytoplasmic membrane, the periplasmic space or intracellular contamination. On the other hand, in a number of studies, the mannoprotein component of the yeast CW was investigated [40,41]. The schemes of the yeast CW structure were formal and demonstrated only a possible arrangement of the mannoprotein and glucan layers and areas enriched with chitin in the lateral CW and in the budding zone [49,50], without taking into account the various functions performed by this organelle. There was almost no understanding of how polymers synthesized inside the cell and on the cytoplasmic membrane contribute to the assembly and formation of a unique molecular ensemble of the cell wall located outside the cytoplasmic membrane and separated from it by the periplasmic space. In part, such a situation could be explained by an insufficient level of knowledge about the structure, the mode of attachment and the functions of proteins that make up the yeast cell wall. 

The first evidence of the important role of proteins as a structural element was data on the lysis of cell walls by proteolytic enzymes [51], after which information confirming that the yeast cell wall contains enzymes—most of which are hydrolases—began to appear in [52]. Nevertheless, for a long time, the mechanism of proteins embedding into the CW was unknown. Initially, the data obtained in the literature were scattered, and often contradictory, since there was no systematic study of yeast cell wall proteins at that time. 

As a result of the series of works from the end of the last century to the beginning of this century [53,54,55,56,57,58,59], CW proteins covalently attached to glucan and chitin were well described, and information about the presence of a large number of so-called non-covalently bound to polysaccharides proteins in the CW started accumulating [60,61,62].

Currently, the literature has accepted the division of CW proteins using the method of extracting them into three groups [8,29,31,61,63].

1. Proteins that can be extracted from cell walls with detergents and thiol reagents when heated form the SEP fraction (SDS-thiol-Extracted Proteins). This group includes proteins that are not covalently bound to CW polysaccharides and can be fixed in the CW with or without disulfide bonds formed with other proteins [61,63].

2. Proteins that can be isolated from the cell wall after removal of the SEP-fraction under the action of glucanases and/or chitinases. These are GPI (GlycosylPhosphatidylInositol) proteins that are fixed covalently on polysaccharide molecules. Unlike plasma membrane proteins, they lack a lipid component in the composition of the GPI-anchor, in the remainder of which these proteins are connected via β-1,6-glucan to β-1,3-glucan—a major structural polysaccharide of the cell wall. In some cases, GPI proteins of the cell wall can be bound via β-1,6-glucan to a minor polysaccharide—chitin [54,55,64,65].

3. Proteins that can be isolated from the cell wall after removal of the SEP fraction under the action of alkali. These so-called “alkali extractable” proteins are also covalently fixed on polysaccharide molecules. The composition of this group includes PIR proteins fixed by β-1,3-glucan with alkali-labile bond of the gamma carboxyl group of glutamic acid, arising from specific glutamines of repeating amino acid sequences DGQxQ (x—some hydrophobic amino acid residue) [66,67] and proteins fixed by an alkali-labile bond without the participation of PIR repeats [63,68]. These proteins make up the ASL (Alkali-Soluble Linkage) group.

A significant part of the cell wall proteins belonging to the SEP group are enzymes that can be combined into a group of polysaccharide-remodeling proteins. These proteins have enzymatic activity that allows them to hydrolyze and link glucan and chitin molecules, forming a polysaccharide scaffold in the CW [8,29,31]. Considering the fact that the main structural framework of the cell wall—mGC, represented by covalently bound polysaccharides—must be continuously remodeled, namely, an increase during cell growth, dramatically rearrangement during division and changes in yeast growth conditions, the role of these enzymes in the biogenesis of the CW and their presence in very large amounts in the composition of the CW is obvious. mGC is a large and relatively rigid polysaccharide structure on which proteins are fixed at one (as in the case of GPI) or several (possibly as PIR) points, which also gives some flexibility to the structure of these blocks.

## 3. SEP Proteins Are a Heterogeneous Group of Proteins of the Cell Wall

Depending on the extraction method, SEP fraction proteins can be incorporated into the CW with or without forming disulfide bridges with proteins covalently bound to polysaccharides. It includes proteins bound by disulfide bridges between their molecules and proteins covalently attached to polysaccharides and also proteins which can be extracted in non-reducing conditions. Most of their molecules can apparently provide dynamic remodeling of the yeast cell wall and possibly bind individual molecules of polysaccharide–protein blocks and/or whole blocks together (Table 1). It should be particularly noted that works accumulated in the literature make it possible to select a group of truly non-covalently attached proteins (NCAp) listed in Table 1, first of all Bgl2, besides some of them also have covalent linkage variants, for example, proteins of Gas and Crh families and other [8].

A significant number of papers have been devoted to this group of proteins [26,27,69,70,71,73,74,82]. They can be extracted from the CW without the use of reducing agents only under a high-temperature or Tris solution with an alkaline pH value, or guanidine hydrochloride. Many of these proteins can also be found in vesicles migrating through the CW.

In this review, attention will be focused on these proteins, their functions and their mode of attachment and compartmentalization in the CW.

## 4. Extracellular Vesicles as a Mode of Compartmentalization of NCAp

Recently, there have been increasingly more studies of extracellular vesicles in yeast [9,11,12,85,86,87]. The phenomenon of vesicular transport from the cell to the environment has been known for a long time, for example, in mammalian cells [88] that do not have a rigid polysaccharide shell similar to yeast cells. To date, extracellular vesicles (EV) have been described in almost all microorganisms in which they have been searched for, from bacteria [89] and archaea [90] to fungi [91], in particular, yeast [9], which suggests that EV are an essential component of every living cell [92,93]. Extracellular vesicles with a diameter of 30 to 150 nm are released into the periplasmic space from the cell through the fusion of their multivesicular bodies (intracellular vesicle clusters) with the plasma membrane by exocytosis [94,95].

The question of how EV are transported through the cell wall remains open. According to the idea of the CW as a plastic structure capable of large-scale dynamic rearrangements, there are three possible explanations for this [92,95,96]: (1) EV can pass through the pores of the CW under the action of turgor pressure after leaving the plasma membrane; (2) EV can move through the CW through channels; (3) enzymes that remodel the CW help transport EV through the CW.

The first hypothesis is supported by the size of the pores of *S. cerevisiae*, which can reach 400 nm under stress [97], which is comparable to the diameter of EV. The second hypothesis about guiding channels is supported by the detection of actin and tubulin in many CW preparations [98,99]. The third hypothesis is based on studies of the identification of single and multiple vesicle-like particles directly in the cell wall by electron microscopy methods without detecting any visible channels around the vesicles or changes in a CW structure surrounded by vesicles [100]. It has also been shown that liposomes with a diameter of 60 to 80 nm penetrate through the CW from the outside and reach the plasma membrane in an intact form [93].

We assume that in all possible explanations, glucan-remodeling enzymes, Bgl2 molecules in particular, can play an important role in the advancement of vesicles through the CW. These enzymes, which are localized on glucan fibrils, bind them into a dense structure that can relax due to changes in their conformation, facilitating the advancement of vesicles from the inside out.

The above, in our opinion, convincingly demonstrates the possible importance of non-covalent interactions in the formation and functioning of a complex multicomponent and multifunctional ensemble of a yeast CW. 

Based on data published by researchers who analyzed the contents of vesicles transported through the *S. cerevisiae* CW to the environment [11], we assume that such proteins as Bgl2, Scw4, Scw10, Scw11, Eng1, Pho3, Sun4, Sim1, Uth1, Exg1, Gas1, Gas3, Gas5, Cwp1, Crh1, Crh2, Ygp1, Tos1, Ecm33, Tip1, Pry3, Pst1, Ccw12, Ccw14, Pir1, Hsp150, Pir3 and Cis3 can be considered proteins that may have non-covalent interactions with CW components. These vesicles also include Bgl2. Of these, polysaccharide-remodeling enzymes besides Bgl2 are Scw4, Scw10, Scw11, Eng1, Sun4, Sim1, Uth1, Exg1, Gas1, Gas3, Gas5, Cwp1, Crh1 and Crh2. It should be noted that many of the listed proteins, for example, Scw4, Gas1, Gas3, Gas5, Cwp1, Crh1, Crh2, Tos1, Ecm33, Tip1 and Pry3, can be characterized as double-binding proteins, but the fact that they are found in the fraction of truly non-covalent proteins is beyond doubt.

Although these vesicles were isolated from the yeast growth medium, there is no doubt that before entering the culture medium, they represent a component of the cell wall and contain a complex of enzymes necessary for its remodeling. Due to their looseness in the thickness of the CW, the proteins that make up the contents of these vesicles can be “dropped” in the right amount into any zone of the CW where their presence is currently required. The presence of this group is confirmed by proteins that we found to be extracted into water when heated after the removal of lipids from the CW. In this fraction of proteins were identified Bgl2, Scw4, Scw10, Eng1, Pho3, Ygp1, Tos1, Pry3, Hsp150 [74], Gas1, Gas3, Gas5, Cwp1, Sag1, Crh1, Crh2, Ecm33, Tir1 and Tip1 [73,74].

The question of the possibility of cross-binding through disulfide bridges of proteins belonging to the NCAp group remains completely unclear and open.

It seems likely that the molecules forming the surface of the vesicles while passing through the CW thickness do not form covalent bonds with its CW components.

We tend to consider these vesicles to be another element of the system of non-covalently bound compounds that plays an important role in the remodeling of *S. cerevisiae* CW. The same opinion is shared by the authors of [11], who described these vesicles in detail. It should be noted that the proteins listed above are also found in the extracellular vesicles of *Candida albicans* [9], *Candida tropicalis* and *Candida parapsilosis* [86], while Bgl2 is present in all four yeast species. The set of other proteins varies depending on the type of yeast studied.

Special attention needs to be paid to the presence of the acid phosphatase enzyme (Pho3) in the list of proteins contained in the vesicles [11]. This enzyme was detected by us in the fraction of proteins isolated from CW after their washing with 1% SDS, through incubation in Tris [74,82]. The complex of these data allows us to confidently claim that this enzyme is fixed in the CW without the participation of covalent bonds with both the polysaccharide component and with the help of disulfide bonds with other proteins covalently fixed on the polysaccharides.

In some cases, the extracellular vesicles contain polyP. Their role in them has only recently begun to receive attention. For example, polyP associated with extracellular vesicles contributes to prothrombotic effects in mammals [101]. Another example is the likely involvement of polyphosphates in the formation of extracellular vesicles in the *Candida* yeast consuming hexadecane. The yeasts *C. maltosa*, *C. albicans* and *C. tropicalis* form extracellular vesicles when grown on hexadecane as the sole carbon source [102]. Electron-scanning microscopy showed that these vesicles were associated with the cell wall and may be biofilm components. The presence of polyP in these vesicles was demonstrated by fluorescence microscopy with DAPI, a fluorochrome which shows orange fluorescence with these polymers. The treatment of hexadecane-grown cells with yeast Ppx1 polyphosphatase led to the degradation of vesicles, observed by scanning and fluorescence microscopy, and the release of orthophosphate into the medium [102]. These results indicate the important role of polyP in the formation of extracellular vesicles in the *Candida* yeast consuming hexadecane. 

## 5. Polyfunctional Protein with Amyloid Properties, Bgl2, Is the Most Studied NCAp

The first and most studied CW protein of many yeast species for which the absence of covalent bonds with both polysaccharides and other CW proteins covalently fixed on polysaccharides is convincingly shown is the glucan-remodeling protein glucanosyltransglycosylase Bgl2, which is a major CW protein abundantly represented in this compartment.

In 1989, Klebl and Tanner, studying the mannoproteins of the cell wall of yeast *S. cerevisiae*, isolated a thermostable protein whose molecular weight, in accordance with its mobility in PAGE under denaturing conditions, was 29 kDa [69], and therefore, it was named gp29. Treatment with endo-β-N-acetylglucosaminidase F revealed the presence of a 3 kDa N-linked oligosaccharide. The authors found lectin-like properties in the protein: whether glycosylated or deglycosylated, it had a high affinity for glucan and chitin, but did not bind to cellulose or starch. With the help of a yeast genomic library embedded in the plasmid vector, the authors carried out molecular cloning. The selection of clones giving a positive signal for hybridization with labeled oligonucleotides synthesized on the basis of the amino acid sequence of eight peptides obtained as a result of the hydrolysis of purified gp29 was carried out. The corresponding regions were sequenced, and the authors named this gene *BGL2*. The open reading frame contained 313 amino acids with a typical N-terminal signaling peptide which is 23 amino acids long, directing this protein into the cell wall [69]. Computer analysis of the amino acid sequence of gp29 revealed significant similarities with β-glucanases in two plants (barley *Hordeum vulgare* and tobacco). The protein was incorrectly characterized as β-1,3-exoglucanase. The authors noted a difference between the calculated molecular weight (33.5 kDa) and the resulting SDS electrophoresis, but could not explain this phenomenon.

In 1993, Mrsa co-authored with Klebl and Tanner [103] recognized the fallacy of previous studies and identified this protein as endo-β-1.3-glucanase. The optimum pH of endoglucanase activity was in the range of 4.5–6.0. The authors showed that the lowered molecular weight is not the result of post-translational modifications, but is explained by the abnormal migration of the protein into SDS-PAGE under these conditions. Of the two potential N-glycosylation sites—N202 and N284—only the latter is glycosylated [103]. This fact is still not generally accepted, since by now, there have been data on the glycosylation of N202, in contrast to N284 [74,104].

Since the mid-1990s, systematic studies of Bgl2 and its role in the formation of the molecular structure of the yeast cell wall have been conducted.

The Bgl2 protein can be assigned to the family of 17 glycoside hydrolases mainly based on the results of the analysis of the primary structure. To date, a number of studies have been conducted on the enzymatic activity of Bgl2 in vitro, while the activity of a protein isolated directly from the cell wall has been studied in detail in only one of them [70]. It should be noted that Bgl2p in that study was isolated when heated to 70 °C, which somewhat reduces the value of the data obtained, despite the fact that Bgl2 is considered a thermostable protein.

Goldman and colleagues conducted in vitro studies that showed that at low concentrations of glucose oligosaccharides, endoglucanase activity is observed, and at higher concentrations, glucosyltransferase activity prevails [70].

It is reliably known that Bgl2 cleaves the diglucose unit from the reducing end of one of the β-1,3-glucan molecules (the so-called donor chain) and attaches it to another β-1,3-glucan molecule (acceptor chain), setting a β-1,6 bond. In this case, a branched product is formed, and the restoring end of the donor chain is blocked.

It was shown that Bgl2 catalyzes the transglycosylation reaction according to the following scheme:

E + rGm → E*Gm-2 + rG2

E*Gm-2 + rGn → E + rGm + n-2,

where E is an enzyme, rGm(n) is a reducing laminarioligosaccharide with a length of m(n) residues, Gm is a donor (m ≥ 5), E*Gm-2 is an intermediate enzyme complex with a β-1,3-glucan chain, G2 is a released disaccharide, Gn is a glucan acceptor chain (n ≥ 4), and Gm + n-2 is a transferase reaction product in which β-1,3-glucan chains are connected by a β-1,6–bond [70,105]. Transferases from *Aspergillus*, *Candida* and *Saccharomyces* use G5 as a minimal donor substrate [106]. Interestingly, the homologue of the Bgl2 protein in *Tetrapisispora phaffii* yeast (*Saccharomycetaceae* family) is secreted into the environment and exhibits killer toxin activity: it causes ultrastructural changes in the cell wall of fungi of *Hanseniaspora*/*Kloeckera* due to β-1,3-glucanase activity, which limits their proliferation [107].

To date, Bgl2 has not been crystallized, but its structure was nevertheless predicted using the Swiss Model server (https://swissmodel.expasy.org/repository/uniprot/P15703 accessed on 14 February 2024) in an article by Sabirzyanov and co-authors [108]. The structure of this protein predicted in this way is a (β/α)_8_ TIM-barrel. With the help of molecular modeling, the conformation of a left-handed polyproline-II helix was detected in the C-terminal fragment of its molecule [108], which can serve as a potential protein–protein interaction site [109]. The deletion of nine amino acid residues of the C-terminal region of Bgl2 disrupted the incorporation of Bgl2 into the cell wall and led to the release of this protein through the cell wall into the culture media, and it lost its ability to form fibrils, which could be observed when Bgl2 was released into the culture medium of the *ssu21*/*mcd4* strain with a disrupted gene encoding one of the proteins of the GPI-anchor synthesis pathway [27]. In the CW of this strain, the Bgl2 protein was not fixed [110].

In the primary structure of Bgl2p, the potential amyloidogenic amino acid (PAD) sequences F_83_TIFVGV_89_, S_168_WNVLVA_174_ and N_190_AFS_193_ predicted in the primary structure of Bgl2 by the computational algorithms FoldAmyloid, Galzitskaya, AGGRESCAN, DHPRED, Waltz, PASTA and TANGO were identified [27], and the peptides A_80_EGFTIFVGV_89_, V_166_DSWNVLVAG_175_ and V_187_MANAFSYWQ_196_ containing these PADs were synthesized. It was demonstrated that these peptides have the ability to form fibrils at pH values from 3.2 to 5.0. The peptides A_80_EGFTIFVGV_89_ and V_187_MANAFSYWQ_196_ (but not V_166_DSWNVLVAG_175_) could fibrillate at pH values from 5.0 to 7.6. For the full-sized Bgl2 protein, the ability to form fibrils at weak acidic and neutral pH values as well as a loss of the ability to fibrillate with increasing pH values was shown. As a result of this study, it was suggested that the ability of Bgl2 to form fibrils apparently depends on the contribution of sites containing PADs [27].

Morphologically, Bgl2 fibrils differ depending on the method of protein extraction and its localization. Bgl2 isolated from CW without trypsin treatment forms long (over several tens of microns in length) fibrils and clusters similar to “bundles” or “nests” with obvious aggregation centers [27]. At pH 5.0, when Bgl2 was isolated from trypsin-treated CW, the fibrils had curved, worm-like forms and were shorter (up to 10 microns long). Fibrils of this morphology were characterized earlier for well-known amyloid fibrils: “nests” for glucagon fibrils [111], and worm-like ones for Aß fibrils [112]. In the culture medium, Bgl2 formed fibrils of the “network” type [27], which differ from the fibrils formed by Bgl2 extracted from the CW.

It is known that under conditions of sherry aging accompanied by ethanol stress, *S. cerevisiae* yeast (flor yeast strain) is capable of forming biofilms [113,114]. In this process, the important role of the Bgl2 protein was demonstrated, while it was also shown that some of the Bgl2 molecules changed localization and were detected in the growth medium [34]. The role of Bgl2 in biofilm formation has also been shown in *C. albicans* yeast [115].

The function of the adhesive for Bgl2 has been demonstrated in several studies conducted on *C. albicans* culture [116,117].

The presence of Bgl2, the molecules of which are extracted from the CW under different conditions and differ in posttranslational modifications in the CW, occurs in the form of several pools [74]. Regulation of the functioning of non-covalently bound glucanosyltransglycosylases that have to remodel mGC to provide CW extension is poorly understood. We demonstrated that Bgl2 and Scw4 have phosphorylated and glutathionylated residues and are represented in the CW as different pools of molecules with various levels of firmness of attachment. The identified pools contain Bgl2 molecules with unmodified peptides, but differ from each other in the presence and combination of modified ones, as well as in the presence or absence of other CW proteins. In combination with its amyloid properties [26,27] and its functions, this indicates a possible role of Bgl2 as an adhesin. The ability of this protein to change localization from cell-wall to extracellular [113,114], along with its strong fixation on CW polysaccharides, may indicate its conformational lability, allowing this protein, non-covalently fixed in the matrix of the cell wall, to perform numerous functions in the changing conditions of yeast growth and make it one of the most noticeable and functionally significant non-covalently fixed proteins of this compartment from the NCAp groups.

## 6. Non-Covalently Bound CW Polyphosphates Are a Necessary Component of the Cell Wall and Possible Participants in Enzyme Activity Regulation 

High-molecular polyP is a minor but very functionally significant component of the CW [118,119,120,121,122]. Evidence in favor of the existence of specific fractions of inorganic polyphosphate in the cell wall of fungi has long been available [47]. PolyP was revealed outside the plasma membrane of *Kluyveromyces marxianus* through the fluorescence of DAPI (4′6-diamidino-2-phenylindole) and X-ray microanalysis [120,123]. There is a correlation between the accumulation of the polyP4 fraction and cell wall polysaccharides in *S. cerevisiae* [4,5,124,125]. This correlation was explained by the presence of dolichyl-diphosphate:polyphosphate phosphotransferase activity in the membrane fraction of *S. cerevisiae* cells and the transfer of phosphate residues from DolPP (dolichyl pyrophosphate) to polyP chains localized in the ER (endoplasmic reticulum) lumen [125,126]. The essential role of dolichyl phosphate (DolP) as a carbohydrate carrier during protein *N*-glycosylation is well established [8]. Some part of the DolP pool is derived from the recycling of DolPP (dolichyl pyrophosphate) after each cycle of *N*-glycosylation, when the oligosaccharide is transferred from the lipid carrier to the protein and DolPP is released, and then, dephosphorylated [8]. However, the gene responsible for dolichyl-diphosphate:polyphosphate phosphotransferase activity has not been identified. More evidenced were obtained in favor to the synthesis of cell wall polyP by Vtc4 polyphosphate synthase. In the ER membrane, a Vtc4/Vtc2/Vtc1 complex was found [126] which can provide polyP in ER lumen. The phosphates needed for polyP synthesis are probably derived from the dephosphorylation of DolPP by the Cwh8 protein, a dolichyl pyrophosphate phosphatase with a luminal-orientated active site [127]. 

The functions of cell wall polyP are related to the ability of these negatively charged polymers to interact with polysaccharides and proteins of this cell compartment. PolyP are responsible for maintaining a negative charge on the cell surface of fungi [121,122]. 

Anionic polyP is able to bind with positively charged groups directly and negatively charged groups indirectly, due to complexes with metal ions. For example, proteins with consecutive histidine residues undergo histidine polyP modification. This polyP modification is non-covalent and involved in protein activity regulation [128]. Another example of the direct binding of polyP which affects protein activity is the participation of this molecule in the regulation of Bgl2 activity; there has also been a proposed hypothetical scheme of this regulation [70]. Moreover, polyP is an inorganic polyanion with protein-like chaperone qualities [129], which can play an important role in the process of the protein–protein interaction [130,131], and also a physiological modifier that accelerates amyloid fiber formation [132]. 

Metal binding by the yeast cell wall has been known for a long time [133]. The metal–polyP complexes are well characterized [134]. Specific DAPI fluorescence indicated an increase in polyP levels in the cell walls of many yeast species exposed to toxic concentrations of heavy metal ions [135]. 

PolyP play an important role in the structural organization of the cell wall. In *C. maltosa* consuming hexadecane as a sole carbon source, special supramolecular structures, so-called “canals” carrying specific polysaccharides and oxidative enzymes, were observed in the cell wall [136]. These structures were revealed by transmission electron microscopy and scanning electron microscopy. No canals were found in the cell wall of *C. maltosa* grown on glucose. The cell wall of hexadecane-grown *C. maltosa* fluoresces more intensively with DAPI than that of glucose-grown cells. This fluorescence disappeared after a treatment with polyphosphatase Ppx1 [102]. The presence of polyP in the cell wall of the pathogenic yeast *Cryptococcus neoformans* was demonstrated by combining fluorescence microscopy, biochemical extraction, scanning electron microscopy, electron probe X-ray microanalysis and the 3D reconstruction of high-pressure-frozen and freeze-substituted cells by focused ion beam-scanning electron microscopy [137]. The Δ*vtc4* mutant lacking the polyphosphate synthetase and a triple mutant lacking PHO transporters demonstrated changes in the cell wall architecture, which was revealed by multiple microscopic approaches [137].

Summing up, the functions of these polymers can be characterized as regulatory and protective, since these polymers are involved in regulation of the activity of enzymes, as well as in maintaining a negative charge on the surface and neutralizing substances toxic to their cells by forming complexes with them.

It has been shown that Pho3 is responsible for the hydrolysis of high-polymer polyP on the surface of yeast cells. An increase of the level of high-polymer polyP in the CW of the Δ*pho3* strain compared with the parent strain was demonstrated [82]. It was found that the chaperone function of polyP is chain length-dependent, with longer polyP chains being disproportionally more effective in preventing protein aggregation in vitro than short-chain polyP [130]. Also, a comparative analysis of CW proteins showed the presence of proportional dependence of the extractability of acid phosphatase on the amount of extracted Bgl2, and revealed a change in the mode of attachment of Bgl2 in a CW strain devoid of Pho3 [82]. In the absence of Bgl2, acid phosphatase, apparently, can hydrolyze CW polyphosphates with a lower intensity, which manifests in their accumulation [82]. It has been shown that high-polymer polyP can participate in the activation of Bgl2 [71]. This indicates another fact, demonstrating the mutual influence of Bgl2 and acid phosphatase on the CW. 

We assume that Bgl2 and Pho3, two important components of the pool of molecules embedded in the cell wall without forming covalent bonds, are capable of forming a metabolon [82] that combines the biogenesis of the main structural polymer of CW—glucan—and the most important regulatory polymers are polyphosphates [47,138]. This hypothesis implies that polyphosphates undergo cyclic transformations in the yeast cell, entering the CW in the form of high-polymer molecules, where they interact with glucan-remodeling enzymes, primarily with Bgl2, after which the acid phosphatase Pho3 hydrolyzes them to orthophosphate, which comes back into the cell and enters the metabolism again. Thus, these molecules are involved in the regulation of glucan remodeling but do not accumulate in the CW. An increase in the content of polyphosphates in the CW leads to a change in the distribution of Bgl2 in this organelle [82], and the incubation of CW containing Bgl2 in the presence of polyphosphates causes an increase in the transferase activity of this enzyme [71]. The effect of polyphosphates on other glucan-remodeling enzymes has not been studied yet.

Post-translational modifications of glucan-remodeling enzymes are another possible way to regulate their activity in the CW [74]; however, a direct connection with the non-covalent attachment of enzyme molecules participating in such modification has not yet been found.

**Figure 1:**
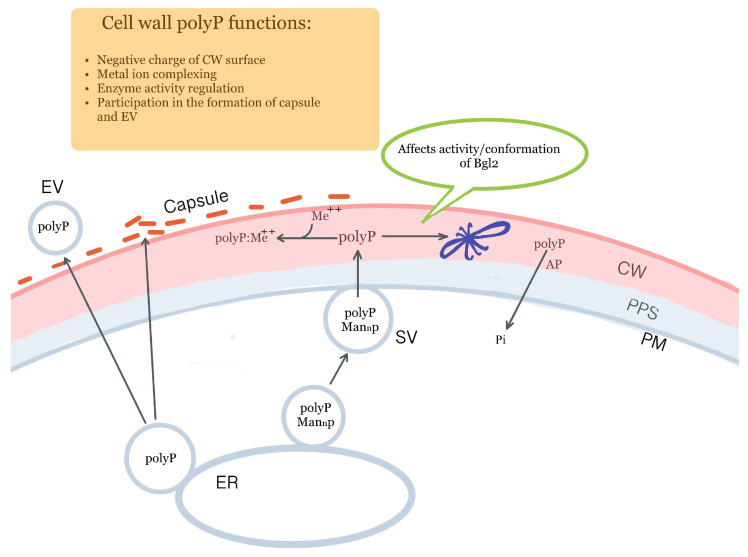
Functions of inorganic polyphosphates on yeast cell surface: hypothetical scheme. PolyP—polyphosphates, CW—cell wall, PPS—periplasmic space, PM—plasma membrane, EV—extracellular vesicles, SV—secretory vesicles, ER—endoplasmic reticulum, AP—acid phosphatase Pho3, Bgl2—blue bow, Pi—orthophosphate, Man_n_p—mannosylated proteins, Me^++^—divalent metal ions. Some yeast have a capsule on their surface, which is depicted on part of the CW surface.

A simplified scheme of polyP functioning in the yeast cell envelope is shown in Figure 1.

## 7. Conclusions

Glucan- and chitin-remodeling activity has been demonstrated for many enzymes, which in some cases leads not only to elongation, but also the branching of glucan molecules and their attachment to mGC. Considering that glucan represents most of the mass of mGC [2] and is its main structural polymer, along with a minor component, chitin [2], their remodeling is a central process that constantly occurs during growth, division and the response to changes in the environment. Thus, it can be confidently stated that the enzymes involved in remodeling are key participants in the formation and functioning of the multicomponent and polyfunctional ensemble of the CW [139,140,141]. 

Characterizing the mode of attachment and functioning of these enzymes, it should be noted that they are localized in direct contact with their substrate (polysaccharides) and are deprived of the possibility of movement along the cell surface inside the cell wall. 

Precise regulation of the activity of glucan- and chitin-remodeling enzymes is vital for the cell, since the uncontrolled activation of these enzymes can possibly lead to destruction of the cell wall and autolysis of the cell, whereas insufficient activity of these enzymes will inevitably affect the growth rate and formation of the CW. Thus, these enzymes should be distributed throughout the cell wall, in which an increase in the cell surface is expected during the growth of mGC, or its destruction and formation anew (in the zones of division and budding). At the same time, polysaccharide-remodeling enzyme activation should occur only where and when it is necessary. Characterizing the described situation as a whole, we use the term «paradoxes» of yeast cell wall polysaccharide-remodeling enzymes. The resolution of these paradoxes can be facilitated by the non-covalent fixation of these enzymes and their interaction with the non-covalently stored cell wall polyphosphates and enzyme Pho3 responsible for regulating their content in this structure. Thus, PolyP are involved in the regulation of enzyme activity and formation of the cell wall. Also, PolyP are important for capsule architecture, the maintenance of negative surface charge and complexation with metal ions.

## 8. Future Directions

Considering the importance of research aimed at studying the mechanism and methods of regulating the formation of the molecular ensemble of the yeast CW from both fundamental and medical points of view, especially taking into account the significant increase in the number of candidiasis in humans and animals, very promising areas could include the following:

− Studying the properties (especially amyloidogenic properties) of NCAp which allow them to be incorporated into the yeast CW so strongly but flexibly;
− Studying the localization and compartmentalization of NCAp in the yeast CW;
− Studying of the role of polyphosphates in the regulation of the activity of enzymes with glucan-remodeling activity;
− Studying of post-translational modifications of yeast CW proteins, primarily polysaccharide-modeling enzymes belong to NCAp in the CW.

## Figures and Tables

**Table 1:** SEP group in *Saccharomyces cerevisiae* cell wall.

Name of Protein	Participating in CW Remodeling	References
Bgl2	+	[26,27,69,70,71,72,73,74]
Scw4	+	[62,68,74,75]
Scw10	+	[62,74,75]
Scw11	+	[62,75]
Sun4	+	[76,77]
Uth1	+	[76]
Sim1	+	[76]
Exg1	+	[26,78,79]
Dse2	+	[77]
Eng1/Dse4	+	[74,77,80]
Cts1	+	[80,81]
Pho3	hypothetically	[71,74,82]
Ygp1	−	[74]
Tos1 *	−	[74]
Pry3	−	[74]
Hsp150	−	[74,83,84]
Gas1	+	[59,72,73,74]
Gas3	−	[8,73,74]
Gas5	+	[8,73,74]
Cwp1	−	[73,74]
Sag1	−	[73,74]
Crh1	+	[8,73,74]
Crh2	+	[8,73,74]
Ecm33 *	−	[73,74]
Tir1	−	[73,74]
Tip1	−	[73,74]

Participation of protein in CW remodeling, «+»; data on participation in remodeling was not found, «−»; indirect evidence of participation in remodeling, “hypothetically”. For proteins, marked «*», for non-*Saccharomyces* yeast species the participation in CW remodeling have been demonstrated.
